# Design and Methods for a Comparative Effectiveness Pilot Study: Virtual World vs. Face-to-Face Diabetes Self-Management

**DOI:** 10.2196/resprot.2415

**Published:** 2012-12-17

**Authors:** Milagros C Rosal, Robin Heyden, Roanne Mejilla, Maria Rizzo DePaoli, Chetty Veerappa, John M Wiecha

**Affiliations:** 1 University of Massachusetts Medical SchoolDivision of Preventive and Behavioral MedicineWorcester, MAUnited States; 2 Heyden Ty, LLCAlameda, CAUnited States; 3 Department of Family MedicineBoston Medical Center and Office of Academic AffairsBoston University School of MedicineBoston, MAUnited States

**Keywords:** Technology, Virtual systems, Education-distance, Patient education, Minority health, Health disparities, African Americans, Type 2 diabetes, Health behavior, Clinical trials

## Abstract

**Background:**

Type 2 diabetes (diabetes) is a serious threat to public health in the United States and disproportionally affects many racial/ethnic minority groups, including African Americans. Limited access to treatment and high attrition rates further contribute to health disparities in diabetes-related morbidity and mortality among minorities. Greater opportunities for increasing access and decreasing barriers to treatment are needed. Technology-based interventions have potential for accomplishing this goal but evidence of feasibility and potential effectiveness is lacking, especially for populations that traditionally have limited educational attainment and low computer literacy.

**Objective:**

This paper describes the design and methods of a pilot randomized clinical trial that will compare the feasibility and potential efficacy of delivering a diabetes self-management intervention via a virtual world vs. a face-to-face format.

**Methods:**

Study participants (n=100) will be African American women with uncontrolled type 2 diabetes recruited from primary care practices and affiliated health centers at a large safety net hospital in Massachusetts. Participants will be randomized into a virtual world-based (VW) intervention condition or a face-to-face control condition. Both conditions provide the same theory-based curriculum and equivalent exposure to the self-management program (eight group sessions), and both will be delivered by a single intervention team (a dietitian and a diabetes educator). Assessments will be conducted at baseline and 4 months. Feasibility will be determined by evaluating the degree to which participants engage in the VW-based intervention compared to face to face (number of sessions completed). Potential efficacy will be determined by comparing change in physiological (glycemic control) and behavioral (self-reported dietary intake, physical activity, blood glucose self-monitoring, and medication adherence) outcomes between the experimental and control groups.

**Results:**

The primary outcomes of interest are feasibility of the VW intervention and its potential efficacy on glucose control and diabetes self-management behaviors, compared to the face-to-face condition. Analysis will use a two-sample Kolmogorov-Smirnov test for changes in variable distribution. *P* values will be calculated using binomial tests for proportions and *t* tests for continuous variables.

**Conclusions:**

If the intervention is found to be feasible and promising, it will be tested in a larger RCT.

## Introduction

Type 2 diabetes (diabetes) is a serious threat to public health in the United States and the world and is a costly disease at the individual and societal levels. In the United States, its prevalence has risen among all ethnic groups, especially among certain racial/ethnic minority groups such as African-Americans who have had a near doubling of prevalence (now 13%) since 1988 [1,2]. Diabetes has numerous and serious health risks associated with poor control [2]. Expert guidelines promote counseling for patient health behavior change targeting increased physical activity, reduction in caloric intake and weight, and promotion of adherence to medication [3]. Among individuals with diabetes, women and African Americans report the lowest levels of physical activity and more than half (66%) of African Americans with diabetes report high-fat diets [4,5]. Although self-management programs have proven efficacy, attrition rates are high [6]. Factors reported as contributing to attrition include competing family responsibilities, conflict with program hours, and distance to services [7]. Web-based methods to improve diabetes outcomes could improve access to behavior counseling and have shown promise in improving health behaviors and glycemic control [8]. However, the feasibility of intervening through web-based methods has been questioned due to a real or perceived digital divide or socioeconomic inequalities among individuals in access to information technologies. For example, only 49% of African Americans compared to 68% of Caucasians have a broadband Internet connection at home [9]. In addition to this divide in access to connectivity and hardware, researchers have identified a skill and knowledge divide on the basis of technological competency and digital literacy [10].

Virtual world (VW) environments are potentially suitable environments for supporting patient education delivery, including diabetes self-management programming. VWs are 3D, immersive, online places where people enter and participate as an avatar. Once in the VW, participant avatars can walk, run, fly, talk, travel, and interact with the other people or structures present in the virtual space. The rich visual landscape, in combination with the mediated presence via avatar, gives the participant a strong sense of “being there”. VWs offer vast opportunities for interaction, intense engagement, and opportunities for scripted immersive experiences, simulations, role-playing, and constructivist learning. Sense of presence, easy access, and anonymity afforded by the avatar may lead to greater interaction and group cohesiveness, as well as engagement and attention, compared to other web-based approaches [11,12]. Currently there are over 300 such online VWs, such as Jibe, OpenSim, Spoton3D, Open Wonderland, and Second Life (SL). Created and maintained by companies, universities, or individuals, some of these online environments are free of charge or some assess monthly or per-use fees. Although the use of VWs has been shown to influence behavior in the “real” world [13], studies are limited and have not targeted the spectrum of diabetes self-management behaviors.

The objective of this paper is to describe the design and methods of a pilot study that will compare the feasibility and potential efficacy of delivering a diabetes self-management intervention via a VW (delivered in Second Life, or SL) versus a face-to-face format.

## Methods

### Design

Women in Control is a pilot randomized clinical trial funded by the National Library of Medicine/National Institutes of Health that will evaluate the feasibility and potential efficacy of a diabetes self-management intervention delivered through interactive sessions within a Virtual World (VW), compared to a face-to-face format, to improve glucose control and enhance adherence to diabetes self-management behaviors in a population of African American women.

### Hypothesis

We hypothesize that the VW-based experimental intervention condition will produce outcomes related to feasibility and potential efficacy that are comparable or greater than those observed in the face-to-face control condition.

### Interventions

The intervention content will be adapted from the CDC/NIH program “Power to Prevent” (P2P) [14], a culturally appropriate, evidence-based behavior-change curriculum designed for face-to-face delivery to African American patients with diabetes or pre-diabetes. The intervention approach is theory-based [15] and thus seeks to enhance diabetes knowledge, optimize attitudes toward diabetes self-management (ie, self-efficacy, outcome expectations), and develop behavioral self-management skills (eg, goal setting, tracking self-management behaviors and glucose levels, problem-solving) to facilitate changes in dietary intake, physical activity, blood glucose self-monitoring, and medication adherence. Curriculum objectives and topics are summarized in Table 1.

**Table 1:** Women in Control diabetes self-management program outline.

Session number	Session topics
1. (Individual)	Intake session—Program overview; Review of informational handouts; Take history of dietary and physical activity habits, medication intake, blood glucose self-monitoring; Discuss action plan and tracking; Set initial goal
2. (Group)	Getting Started—Welcome and introductions; What is diabetes; Diabetes self-management goals; Achieving control through small steps; Ways to increase physical activity
3. (Group)	Decreasing Your Hunger—Food and blood glucose; Healthy food choices from three food groups; Healthy fats; Fiber
4. (Group)	Using Food Labels to Track Carbs and Fat—Finding carbohydrates and fat grams in a food label; Healthy amounts of fiber per serving; Decreasing fat grams in food choices; Substituting healthful foods for less healthful foods
5. (Group)	What Have We Learned So Far?—Review of the effects of physical activity and food on blood sugar
6. (Group)	Diabetes Medications—Benefit of medications in diabetes management; How medications work; Importance of adhering to the medication regimen; Strategies to enhance medication adherence
7. (Group)	Physical Activity for You and Your Family—Review of overall benefits of physical activity for patient and family; Eliciting support for physical activity from others; Relapse prevention
8. (Group)	Managing Food Intake and Blood Sugar Outside of the Home—Understanding the difference between portion size and serving size; Strategies to improve food choices when eating out; Preventing overeating when eating out
9. (Group)	Empowering Yourself—Asking questions of health care providers; Remembering goal levels for A1c, blood pressure, and cholesterol; Graduation

We will deliver this curriculum through a series of weekly sessions that begin with an individual intake session, followed by 8 weekly sessions. Sessions will be delivered in either a VW or face-to-face environment in groups of approximately 12-15 participants by a single intervention team (a dietitian and a diabetes educator) for the same duration (90-minute sessions) using the same protocol and materials for both conditions. Participants in both conditions will receive 2 sessions of computer training and will be provided with an Internet-enabled laptop computer upon training completion. Multimedia Appendix 1 summarizes the specific activities conducted during each computer training.

**Figure 1:**
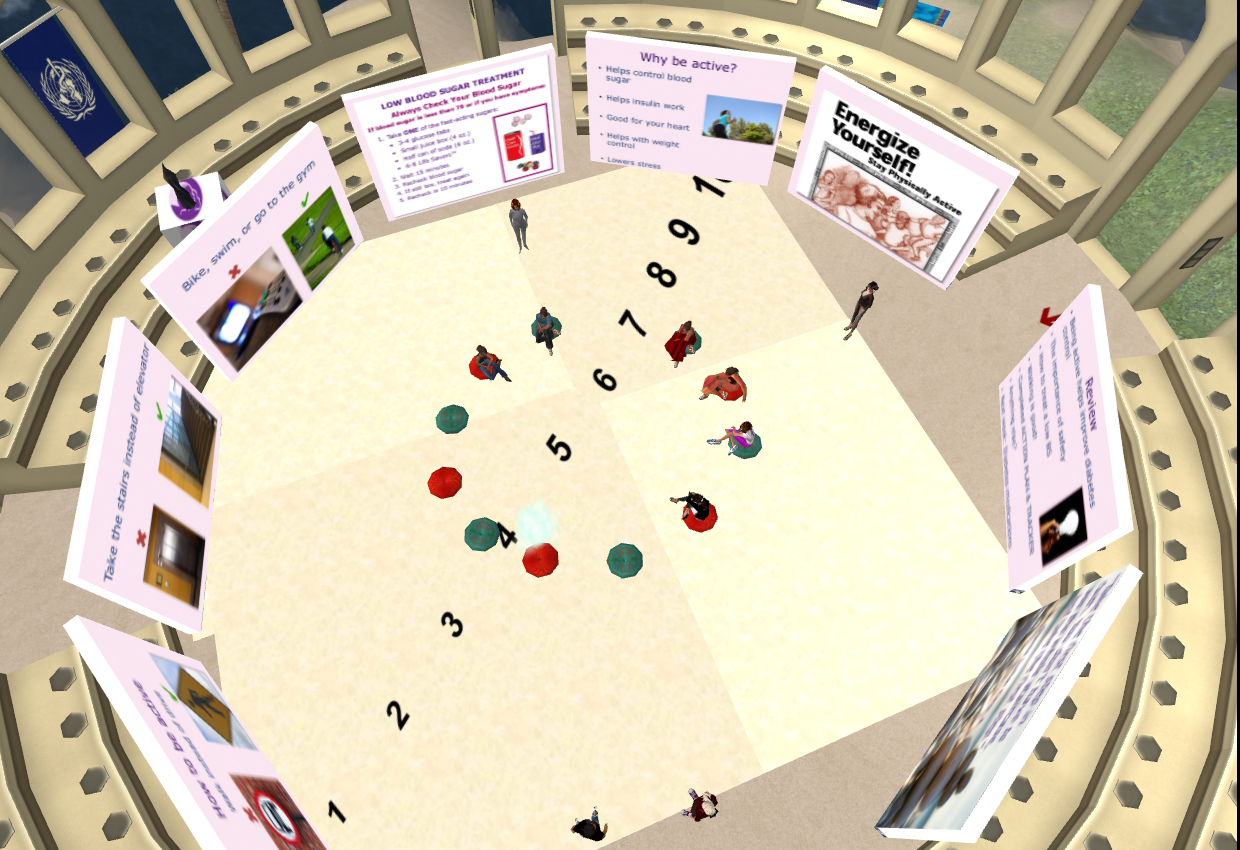
Virtual world forum.

**Figure 2:**
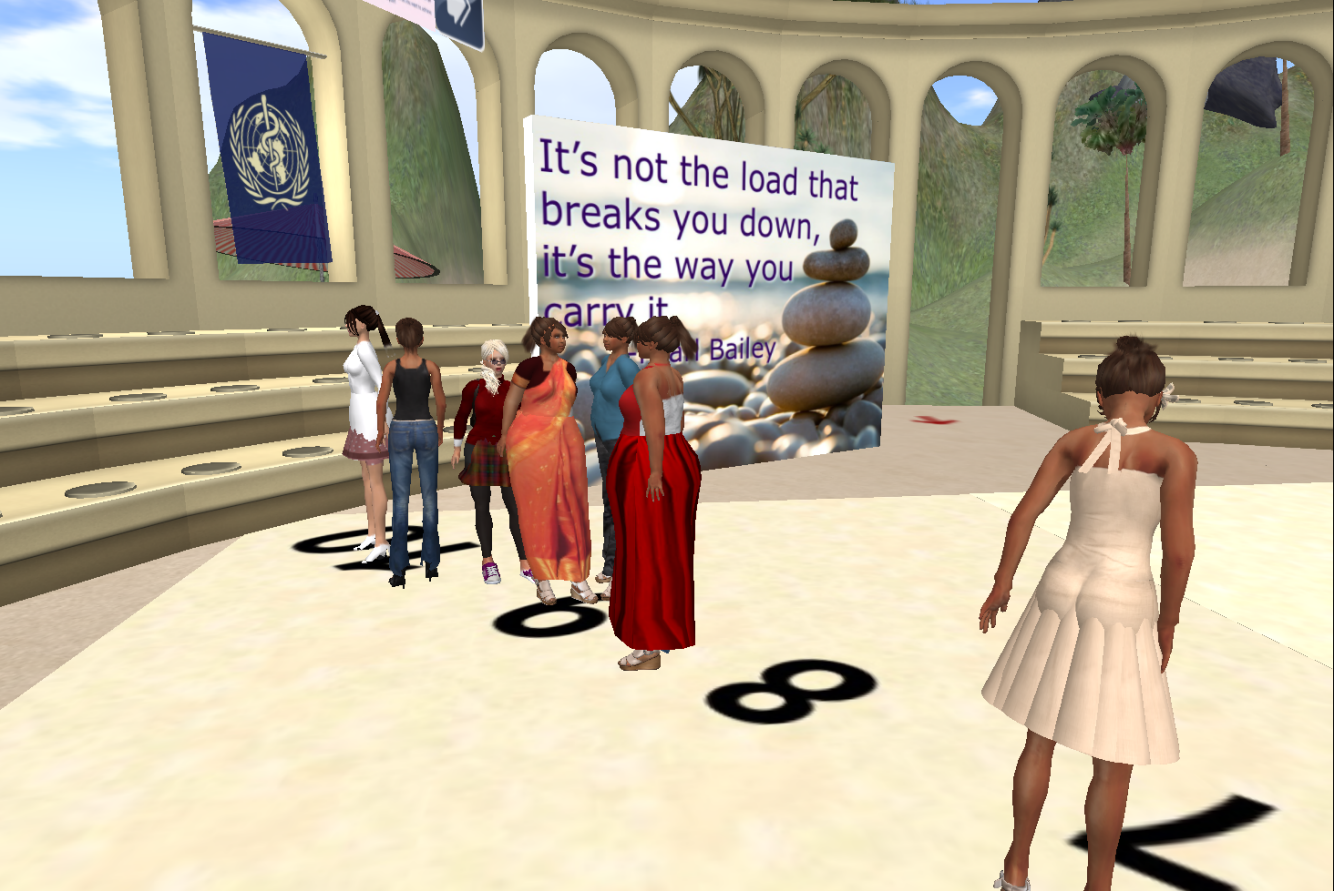
Floor pattern: self-efficacy ruler.

**Figure 3:**
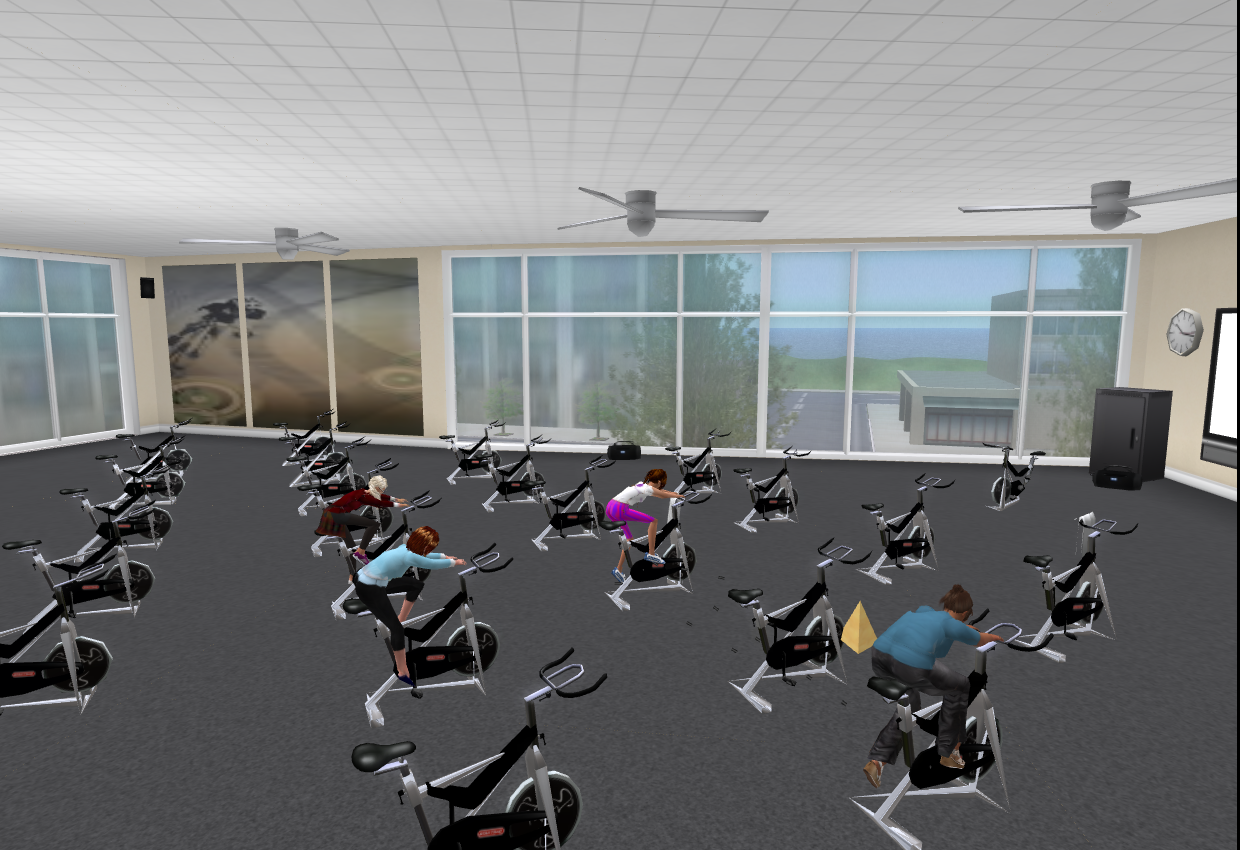
Club one island.

**Figure 4:**
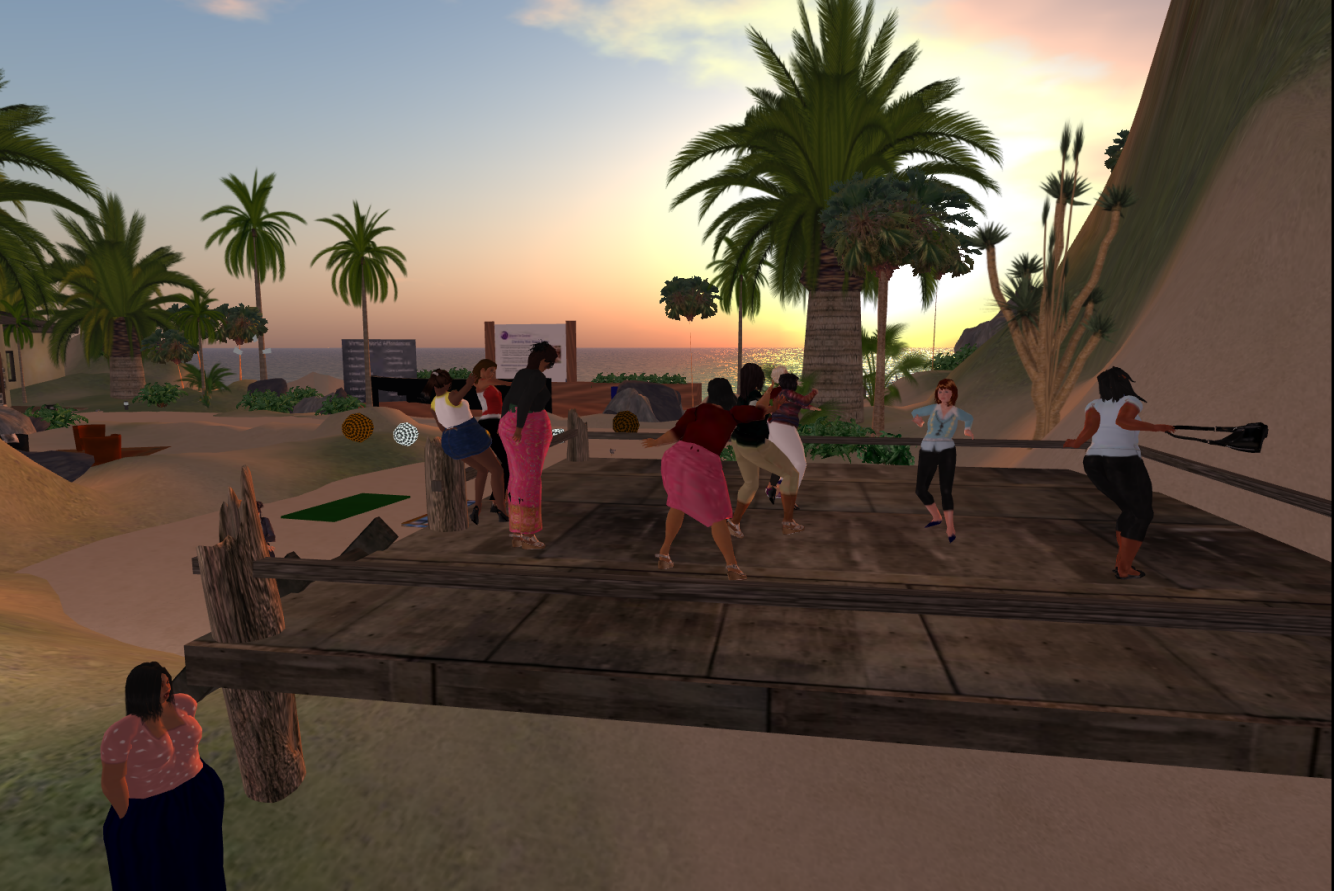
Dancing stage.

The face-to-face group sessions will take place in a large conference room at Boston Medical Center. All face-to-face participants will receive transportation vouchers to facilitate attendance. VW-based sessions will take place in an open-air VW forum built especially for the intervention with adequate structures and required visuals/displays (see Figures 1 to 4). Although the VW sessions will last 90 minutes, these participants will be asked to log in 30 minutes prior to each session in order to test their sound and troubleshoot any connection problems. To facilitate session flow, a triage system will assign participants to “producers” (technical support staff) as technology needs arise. As in the face-to-face condition, both interventionists will attend every group session (alternating facilitation). In the VW condition, the non-leading interventionist will take notes in the local chat and provide general back-up, while in the face-to-face condition, the non-leading interventionist will provide logistical assistance. Multimedia Appendix 1 compares similarities and differences of intervention implementation among these conditions in greater detail.

Two members of the research team will oversee fidelity of intervention content and style for both conditions via direct observation and review of recorded sessions. Interventionists will be provided with feedback on errors of omission and commission, and additional support will be given on an ongoing basis as needed.

### Study Setting and Population

The study will recruit 100 African-American (Afro-Caribbean) women recruited from primary care practices and affiliated health centers at a large safety net hospital in Massachusetts. Eligible subjects will be age >18 years, English-speaking, with a diagnosis of type 2 diabetes, and an HbA1c > 8 at their last outpatient visit, which has to occur within the year preceding recruitment. Subjects with the following conditions will be excluded: ulcerative colitis, renal failure, complications following abortion and ectopic and molar pregnancies, angina pectoris, and other forms of unstable ischemic heart disease and conditions precluding brisk walking.

The research protocol was approved by the Institutional Review Boards at Boston Medical Center and the University of Massachusetts Medical School. Potential participants will be identified from the medical record data warehouse at Boston Medical Center and affiliated community health centers based on the above criteria. Identified patients will receive a letter informing them about the study. The letter will provide a description of the study, announce a phone call from a study staff to determine final eligibility, and give patients the option to call in or opt out. During the telephone call, the staff will assess final eligibility (ie, self-reported ability to view a computer screen without difficulty, ability to read, no use of glucocorticoid therapy, no current participation in a weight loss program, and availability for weekly meetings), answer any questions they may have about the study, and invite fully eligible women to participate. A maximum of five calls will be made to each participant on different days and varying times. Interested women will be scheduled for an enrollment visit at the Boston Medical Center General Clinical Research Unit in which participants provide written informed consent. A reminder call will be made the evening prior to the appointment.

### Randomization

Upon completion of all study assessments, we will randomize participants to either the VW-based experimental intervention condition or the face-to-face control condition. A block randomization scheme with a block size of 4, developed by StudyTRAX software (v3.0.0103), will be used. Randomization will be stratified on age and hemoglobin A1c measured at baseline. Participants will be informed about their randomization assignment via phone.

### Outcomes and Study Measures

The primary outcomes of interest are feasibility of the VW intervention and its potential efficacy compared to the face-to-face condition. Feasibility will be determined by evaluating the degree to which participants engage in the VW-based intervention compared to face-to-face (number of sessions completed). Potential efficacy will be determined by comparing change in glucose control and self-management behaviors between the experimental and control groups, from baseline to the 4-month follow-up.

Assessments will be performed at baseline and at 4-month follow-up. HbA1c tests will be used to assess average blood glucose level over the previous 2-3 months. Non-fasting venous blood samples from an antecubital vein will be collected after the subject remains seated for 10 minutes, in accordance with standard research protocols [16]. Boston Medical Center laboratories will analyze the specimens. At each time point, dietary intake will be assessed by two unannounced 24-hr dietary recalls (24HRs) [17] administered on randomly selected days (60% probability of a weekday and 40% probability of a weekend day). 24HRs are well suited for the characteristics of the target population (ie, language, illiteracy/low educational level, ethnic foods) that limit use of other/additional assessment methods. Also at each time point, 24-hour physical activity recalls will be administered immediately after the 24-hr dietary recalls [18], followed by recalls of blood glucose self-monitoring and medication adherence. Other measures will include blood pressure [19], cholesterol, body mass index, waist and hip circumference, depressive symptoms [20,21], self-efficacy for diabetes management [22], health literacy [23], social support [24,25], perceived stress [26], and quality of life [27]. Demographic factors, smoking history, alcohol intake, current use of prescription and non-prescription medications, as well as use of home remedies, and experience with computers and the Internet will be assessed via survey. We will assess patient satisfaction at the 4-month follow-up. Intervention implementation costs (costs that would be incurred if the intervention were to be implemented outside the context of the research project) will be tracked.

### Data Management

StudyTrax (v3.0.0103), a web-based electronic data capture software, will be developed to house case report forms for data collected throughout the study. Case report forms will be developed to include field-specific validation code, procedures to check data ranges, and reminders to enter required data to ensure data integrity. Research staff utilizing the database will be provided role-specific privileges to the database. Two members of the study staff will perform double data entry on all clinical measures. Dietary data will be collected using the Nutrition Data System for Research (NDSR) database. Physical activity data will be collected on case report forms designed in Microsoft Access.

### Quantitative Evaluation: Analytic Plan

#### Sample Size and Power Calculations

As a comparative effectiveness study, patients in both arms will receive interventions potentially resulting in improvements in the primary outcomes. Hence, a very large sample size would be required to demonstrate either statistically significant comparative effectiveness or superiority of one group over the other. As a result, and in recognition of the project’s status as a feasibility study, the study was powered to be able to demonstrate within group improvements in one of the key outcomes: physical activity. A sample size of 47 was determined to be sufficient to show a within group improvement, from pre- to post-, of 20% of the sample from inactive to moderate intensity physical activity, with a power of 80% at 0.05% level of significance.

#### Analysis and Statistical Tests

Our null hypothesis is that there will be no significant differences in outcome measures between the VW and face-to-face conditions. We will test this null hypothesis against the alternative that the VW condition is not as effective as face-to-face intervention using a two-sample Kolmogorov-Smirnov test for changes distributions of A1c. In other words, the VW intervention will be as good as or better than face-to-face intervention in decreasing A1c. The *P* values will be calculated using binomial tests for proportions and *t* tests for continuous variables. We will use RStudio (version 0.96.330) [28] for statistical analysis.

### Qualitative Evaluation

Following completion of the intervention delivery phase, focus groups will be conducted to qualitatively compare the experiences of participants assigned to the VW vs. the face-to-face condition. A total of 40 randomly selected participants will be invited to participate in four focus groups (expecting that 8-10 participants per group will attend): two groups will include VW participants, and the other two will include face-to-face participants. Participants will be consented for participation in, and recording of, the group discussions. We will offer a monetary incentive of US $40 and travel expenses to encourage participation. Two analysts will independently review transcripts of discussions, identifying key words and themes (in vivo coding) related to their experiences of living with diabetes, decision-making about treatments and lifestyle changes, and involvement in the VW or face-to-face intervention. A grounded theory approach will be used [29]. Constant comparative analysis will be used to relate the transcribed focus group interview data through ideas to core concerns. The first approach will involve open coding using a meaningful phrase by meaningful phrase strategy. During the open coding process, data will be coded for any and all categories. Focused codes will be created for sorting and synthesizing the initial codes to organize the data, using the most significant and/or frequent earlier codes to sift through the data. From focused codes, memos will be generated theorizing about how codes relate to ideas and characterizing the emerging theory. Memos will be sorted into concepts demonstrating relationships between concepts that derive from memos. The formulation of themes and codes drawn directly from different sections of the data, and then used to code the transcripts themselves, is a pivotal step toward building an in-depth analysis.

## Discussion

Glycemic control is critical to preventing health disparities in diabetes morbidity and mortality among racial/ethnic minorities such as African Americans. Strategies to maximize access to treatment and self-management support are needed for this and other vulnerable populations. Technology-based interventions may be able to increase access but further evidence is needed to support the feasibility of such interventions with populations that have limited educational and computer literacy. By comparing a VW-based intervention format to a traditional face-to-face format for delivery of a diabetes self-management intervention to a sample of low-income African American women, this study will provide evidence of feasibility for a population with considerable health disparities in diabetes prevalence and outcomes and who also have limited experience with computers.

The strengths of the study are many. They include: (1) assessment of feasibility of utilizing a VW platform for patient health education, (2) randomization of study participants to a face-to-face condition (ie, traditional approach) vs. a VW environment condition, holding constant the intervention content, (3) recruitment of inner city minority participants thus addressing so called digital-divide issues, (4) standardization of hardware used to access the VW, (5) use of a control group that has similar hardware and training, (6) robust measurement of behavioral outcomes, and (7) inclusion of a qualitative post-intervention evaluation.

Limitations of this study include: (1) the pilot nature of the study. Adequately powering a comparative effectiveness study, given the characteristics of our primary outcomes, will require a larger sample size, beyond the scope of this project, and (2) the study focus is on a subset of the population, namely low-income African American women with uncontrolled diabetes. Thus, the generalizability of our findings to men and to non-underserved populations might be limited.

Evidence of feasibility and potential efficacy is needed so that new interventions can be designed for VW implementation and high reach to diverse populations. Our previous work has demonstrated the ability of VW-based training to change the professional behavior of clinicians [30,31]. We anticipate that the current study will produce valuable data and insights to help guide application of these findings to VW-based interventions targeting not only diabetes self-management but also other chronic conditions and health behaviors, including such critical topics as medication adherence, substance abuse cessation and treatment, individual and group-based mental health treatments, many possible rehabilitative services, and other health services.

## Multimedia Appendix 1

Comparison of virtual world (VW) versus face-to-face implementation of the Women in Control curriculum.

## Multimedia Appendix 2

NIH Reviewers Statements.

## Multimedia Appendix 3

Response to Reviewers Critiques.
