# Supplementary material for: Design and Methods for a Comparative Effectiveness Pilot Study: Virtual World vs. Face-to-Face Diabetes Self-Management
Source: JMIR Res Protoc. 2012 Dec 17;1(2):e24. doi: 10.2196/resprot.2415 (PMC3626161; doi:10.2196/resprot.2415)
Supplement: Supplementary file 1 [file resprot_v1i2e24_app1.pdf]

Multimedia Appendix 1. Comparison of Virtual World (VW) versus Face-to-Face Implementation of the Women in Control Curriculum.

<table>
<tr><th>Virtual World Condition</th><th>Face-to-Face (Control) Condition</th></tr>
<tr><td colspan="2">Intervention Preparation</td></tr>
<tr><td colspan="2">Set-up</td></tr>
<tr><td colspan="2">Both conditions will include the following intervention materials:
Scripted intervention manual
Slide set for each session
Participants’ binder
Pedometer
Calorie King book and exercise DVD</td></tr>
<tr><td>Sessions will be delivered in large, open-air virtual world (VW) Forum (see Figure 1) on Boston University Second Life (SL) island
Simple landscape for ease of navigation and visual appeal
Session space will be outfitted with cushions for sitting and various floor patterns (a confidence ruler, a color chart, and an agreement pie) to support group process. Exercise areas with yoga mats, tai chi balls, and an automated dance floor will be used
Curriculum slides imported and displayed on large screens that ringing Forum outer circle
Individual sessions (session 1) will take place in private chat areas
Avatars will be created for each participant prior to first session. Physical characteristics will be similar to the patient population (African American hair and skin and 70% body fat) and named according to a convention of first name, first initial of last name (eg, MaryS)</td><td>Sessions will be delivered in a large conference room at Boston Medical Center
The room will accommodate up to 15 participants, the interventionists, the display of educational self-management materials, and equipment (ie, a slide projector, easel)
Participants will be seated around a large table that will facilitate their use of intervention binders and note-taking, if desired
Session 1 (individual) will take place in a private office at Boston Medical Center</td></tr>
<tr><td colspan="2">Interventionists and Staff Training and Support</td></tr>
<tr><td colspan="2">A single set of interventionists (a dietitian and a diabetes educator) will:
Receive the same training consisting of motivational interviewing, delivery of the intervention</td></tr>
</table>

<table>
<tr><td colspan="2">protocol (ie, mock sessions for each condition)
Deliver both interventions (take turns in leading the sessions)
Receive ongoing (weekly) oversight and support</td></tr>
<tr><td>In addition to the above training, interventionists receive approximately 20 hours of SL training
Additional technical support staff include:
Instructional Technology and Operations staff: will purchase, inventory, maintain, and support all computers and related equipment

Two Producers: skilled in basic SL, will provide SL technical support and troubleshooting

Lead Producer: overall SL project manager, manage the SL location and coordinate the producers</td><td>No additional staff or training</td></tr>
<tr><td colspan="2">Pre-Intervention Computer Training</td></tr>
<tr><td colspan="2">All participants will participate in two 2-hour computer training sessions (total of 4 hours).
At the end of the second session, all participants will receive a laptop computer to take home and the intervention binders and other materials.</td></tr>
<tr><td>Session #1:
VW participants will be instructed on their laptop kits (MacBook, power cord, headset, 4G wireless modem), the computer (keyboard, trackpad, basic commands), connecting to the Internet, and will receive a passive introduction to the SL environment (using a presentation screen)
Session #2:
VW participants will meet their avatar and will be taught how to use SL, log in, teleport, move, send messages via local chat and IM
VW participants will have a SL skills check
Additional training or home visit as needed</td><td>Session #1:
Face-to-face participants will be instructed on their laptop kits (Dell laptop, power cord, 4G wireless modem), the computer (keyboard, trackpad, basic commands), and connecting to the Internet
Session #2:
Face-to-face participants will be taught how to use email and Skype, set up accounts, and how to use a web browser to search online</td></tr>
<tr><td colspan="2">Typical Session</td></tr>
<tr><td colspan="2">Session 1 will be administered individually lasting approximately 30 minutes.
Sessions 2-9 will be conducted in a group format lasting 90 minutes.</td></tr>
<tr><td>All encounters occur in SL</td><td>All encounters occur in person</td></tr>
</table>

▪ In a typical session, the VW group consists of the participants' avatars and two interventionists, and two producers provide technical support	▪ In a typical session, the face-to-face group consists of the participants and the two interventionists only
▪ VW participants will be asked to arrive 30 minutes early to test their sound and troubleshoot any connection problems	▪ No additional time requirements
▪ The interventionist not leading the session will take notes in local chat and provide general back	▪ The interventionist not leading the session will assist with logistical issues (ie, late arrivals, setting up materials)
▪ Interventionists will lead discussions by walking participants around the perimeter of the Forum to see slides	▪ Interventionists will lead discussions by using slides and an easel
▪ In addition to group discussions and review of materials in the participants' binder, participants will be involved in activities set up for their avatars. For example,	▪ The curriculum will be implemented primarily through group discussions and review of selected materials in the participant's binder
➢ When asked to indicate goal self-efficacy, avatars will stand on a number from 1 to 10 displayed on the floor (see Figure 2)<br>➢ Avatars will take field trips to a restaurant and a health club (Club One Island) to learn about exercise equipment and practice healthy food ordering and eating practices (see Figure 3)<br>➢ At the conclusion of each session, avatars will be invited to participate in some form of physical activity: dancing, tai chi, yoga, or a walk around the island (see Figure 4)	▪ Interventionists will implement a 5-minute physical activity break at each session (ie, stretching demonstration; walking with pedometer)
