# Supplementary material for: Design and Methods for a Comparative Effectiveness Pilot Study: Virtual World vs. Face-to-Face Diabetes Self-Management
Source: JMIR Res Protoc. 2012 Dec 17;1(2):e24. doi: 10.2196/resprot.2415 (PMC3626161; doi:10.2196/resprot.2415)
Supplement: Supplementary file 2 [file resprot_v1i2e24_app2.pdf]

SUMMARY STATEMENT
(Privileged Communication)

PROGRAM CONTACT:
JANE YE
(301) 594-4927
yej@mail.nih.gov

Release Date: **07/25/2009**

Application Number: **1 RC1 LM010412-01**

Principal Investigators (Listed Alphabetically):
ROSAL, MILAGROS C PHD
WIECHA, JOHN MD (Contact)

Applicant Organization: BOSTON MEDICAL CENTER

Review Group: **ZRG1 PSE-J (58)**
Center for Scientific Review Special Emphasis Panel
RFA OD-09-003: Challenge Grants Panel 1

Meeting Date: **07/20/2009**
Council: **OCT 2009**
Requested Start: **09/30/2009**

RFA/PA: **OD09-003**
PCC: **ARCI**
Dual IC(s): **RA, DK**

Project Title: **Virtual World Health Behavior Counseling for Patients with Diabetes**

SRG Action: **Impact/Priority Score: 50 Percentile: 15**
Human Subjects: 44-Human subjects involved - SRG concerns
Animal Subjects: 10-No live vertebrate animals involved for competing appl.
Gender: **2A-Only women, scientifically acceptable**
Minority: **2A-Only minorities, scientifically acceptable**
Children: **3A-No children included, scientifically acceptable**
Clinical Research - not NIH-defined Phase III Trial

Project Year	Direct Costs Requested	Estimated Total Cost
1	372,106	534,982
2	372,997	532,133
TOTAL	745,103	1,067,115

ADMINISTRATIVE BUDGET NOTE: The budget shown is the requested budget and has not been adjusted to reflect any recommendations made by reviewers. If an award is planned, the costs will be calculated by Institute grants management staff based on the recommendations outlined below in the COMMITTEE BUDGET RECOMMENDATIONS section.

1RC1LM010412-01 Wiecha, John

PROTECTION OF HUMAN SUBJECTS UNACCEPTABLE
SCIENTIFIC REVIEW OFFICER'S NOTES

RESUME AND SUMMARY OF DISCUSSION: The investigators propose a pilot randomized controlled trial to evaluate the feasibility and potential efficacy of a theory-driven intervention delivered through interactive sessions within Second Life (SL) to improve Type 2 diabetes mellitus self-management behaviors among African-American women. The reviewers agreed during the discussion that the application is good and likely to have moderate impact. The study is strong including its significance, focus on African-American women, and novel method for health education medium. However there are moderate weaknesses including lack of expertise in Second Life, limitations of physical activity as the primary outcome, no plan for dealing with decreased adherence; insufficient implementation and training plan; and differential incentives for the intervention and control group.

DESCRIPTION (provided by applicant): This application addresses broad Challenge Area (05): Comparative Effectiveness Research and specific Challenge Topic: 05-LM-104. Value of "Virtual Reality" Interaction in Improving Compliance with Diabetic Regimen. Title: VW-HBCPD: Virtual World Health Behavior Counseling for Patients with Diabetes Type 2 diabetes mellitus (T2DM) is a serious public health concern and its prevalence is greater among African-Americans (AA) compared to Caucasians. Increased physical activity (PA), consumption of a healthy diet and medication intake are key to glycemic control and prevention of complications among persons with T2DM. T2DM self-management programs (SMP) have proven efficacy in improving the risk profile among individuals with diabetes, but attrition rates are high due to conflict with family responsibilities, program hours, and accessibility. Virtual reality (VR) environments like Second Life (SL) are potentially suitable for delivering T2DM SMP. However, studies on the use of VR to influence health behaviors are limited and have not targeted T2DM self-management (SM) behaviors. This 2-year pilot RCT will evaluate the feasibility and potential efficacy of a theory-driven intervention delivered through interactive sessions within SL to improve DM SM behaviors. The proposed intervention is based on the CDC/NIH "Power to Prevent" (P2P) culturally appropriate evidence-based behavior-change curriculum designed for face to face delivery to AA patients with T2DM or pre-diabetes. The intervention will target knowledge, self-efficacy, and behavioral capabilities for the targeted behaviors. It includes 2 components delivered within SL: 1) group counseling sessions based on P2P and, 2) individual counseling sessions to reinforcement of intervention messages. Behavioral targets include a reduction in calorie intake, enhanced dietary quality, increased PA, and increased T2DM medication adherence. Subjects (n=100) will be sedentary AA women with uncontrolled T2DM recruited from primary care practices at Boston Medical Center. Participants will be randomized into the VW-HBCPD intervention, or an equivalent exposure to the curriculum delivered face to face, delivered over 8 months in both arms with assessments at baseline and 8 months. The study's primary aims are to: 1. Determine the feasibility of VW-HBCPD by evaluating the degree to which subjects engage in the intervention (number of sessions completed). 2: Determine the potential efficacy of VW- HBCPD, compared to a control group receiving face to face counseling, on increasing PA. Secondary aims are to: 1) Determine the effect of the VW-HBCPD intervention, vs. face to face counseling, on behavioral (calorie, fat, and fiber intake and medication compliance), anthropometric and physiological (weight, BMI, waist circum., blood pressure, lipids, and HbA1c), and psychosocial (self-efficacy and quality of life) outcomes; 2) Determine the cost (per subject) of implementing the intervention; 3) Document baseline values and variance of outcome variables in order to accurately estimate sample size and statistical power in a subsequent RCT. Despite often intensive educational efforts, patients with diabetes commonly mismanage or under-manage their illness despite the known ability of optimal management to reduce complications and morbidity. Interactions between avatars in virtual reality environments such as Second Life are known to influence behavior. This study will explore the effectiveness of an evidence-based behavior change counseling program for patients delivered in the virtual reality environment Second Life, as compared to the same curriculum delivered face to face, among African-American women with poorly controlled type 2 diabetes who are physically inactive.

CRITIQUE 1:
Significance: 5
Investigator(s): 5
Innovation: 6
Approach: 7
Environment: 5

Overall Impact:

Strengths

- High potential impact if successful in promoting behavioral and psychosocial outcomes in the Type 2 diabetes patients, using Second Life compared to face-to-face counseling.
- Focus on a group of patients, African-American women, who may potentially benefit greatly from the intervention.

Weaknesses

- Lack of expertise in actual implementation of Second Life.
- Content of Second Life is not clear; the investigators need to have a clear understanding of what information will be made available and how.
- Aim two greatly depends on Aim 1 succeeding. There is no recourse for when there is decreased adherence to the protocol.
- Implementation and training of participants is not sufficiently addressed.

1. Significance:

Strengths

- A successful implementation could potentially address self-management and counseling in this subgroup of patients that could impact behavioral change.

Weaknesses

- There is limited information on training participants in using Second Life. The digital divide is substantial in this population and might provide a substantial barrier.
- Unclear what the measurable outcomes are. The primary aim focuses on physical activity using a survey. This may be insufficient to assess small differences.
- Since the investigators will also be measuring hard outcomes (weight, blood pressure, glycemic indices), these should be included as primary aims.

2. Investigator(s):

Strengths

- Investigators are well recognized for their expertise in web-based education for patients populations.
- The PI has a good publication record in home-based patient education.
- Collaborators have expertise in patient recruitment, .

Weaknesses

- Levels of effort propsed by investigators are unlikely to be sufficient for scope of project, raising doubts that goals will be accomplished.
- There is not sufficient expertise in the area of Second Life. The research assistants are going to be fully responsible for recruitment and training. They will need to be trained as well.
- Installation of equipment will be done by research assistants. Will they be going to patients' homes to do this?

3. Innovation:

Strengths

- This is another web-based initiative that could effect behavior change.

Weaknesses

- Several web-based initiatives have been developed for diabetics. It is unclear how this one is any different.
- There is no long-term follow-up to assess sustainability, which is a major problem in this population.

4. Approach:

Strengths

- The study will employ a randomized control trial.
- Use of identical computer platforms to perform the study is sound to minimize technical problems.

Weaknesses

- There is no plan for addressing decreased adherence to the protocol.
- Secondary aims (glycemic control, blood presure, weight) need to be included as primary aims.
- It is unclear why only women are included in the study.
- The study seems to be underpowered to detect substantial improvement (in physical activity) using Second Life.
- The analytical plan needs to be addressed for each aim.
- There is insufficient information on how Second Life will be implemented and what content and format will be used.
- There is insufficient evaluation of the Second Life implementation for this domain prior to use in the clinical trial.
- Success of Aim #1 could depend on implementation and many other factors, including the digital divide.
- There is limited information on training, both staff and patients.
- Investigators mentioned that most households have computers. They should not provide participants with free laptops and simply use the computers in the patients' households.
- Inadequate computers in households would present a problem in potentially expanding this project.

5. Environment:

Strengths

- Outstanding scientific environment in the participating institution.
- Strong commitment of support from the participants.

Weaknesses

- Second Life is proprietary and there should be more provision for security of protected health information.

Protections for Human Subjects:

Unacceptable Risks and/or Inadequate Protections

- It is not clear how patient information will be protected when using Second Life

Data and Safety Monitoring Plan (Applicable for Clinical Trials Only):

Unacceptable

- There is no provision for monitoring data safety.

Inclusion of Women, Minorities and Children:

G2A - Only Women, Acceptable

M2A - Only Minority, Acceptable

C3A - No Children Included, Acceptable

- There is no need to include children in this study.

Budget and Period of Support:

Recommend as Requested

CRITIQUE 2:

Significance: 1
Investigator(s): 1
Innovation: 1
Approach: 2
Environment: 1

Overall Impact:

Strengths

- Novel method for health education medium with great potential and little work to date – virtual reality
- Directed to high risk population with an excellent rationale for appropriateness of this intervention medium in this target population.
- Comprehensive, conceptually solid intervention using state-of-the art interventions
- Excellent comparison condition – meets criteria of comparative effectiveness research, so that knowledge gained will be particularly relevant to future value of intervention

- Entire proposal is thoughtfully organized and written, showing in-depth familiarity with the research area and with implementation and behavior change research. .

Weaknesses

- There is a difference in incentives between intervention and control groups – the VR arm gets a MacBook at end of study. This could influence program adherence. Some comparable incentive should be considered for the control arm.

1. Significance:

Strengths

- Web-based self-mgmt support and health education addresses key barriers to translation in low-income urban populations – the difficulties of transportation and access for in-clinic trainings.
- The medium – virtual reality – is relatively untested in health education, but is extremely promising and of great interest.
- The research team is well suited and well situated, with access to an excellent patient population to conduct this pilot evaluation.

Weaknesses

- None identified

2. Investigator(s):

Strengths

- Dr. Wiecha appears to have a substantial career investment in distance learning for physicians and patients using innovative IT approaches to patient support.
- He and Dr. Rosal have worked together on related interventions; Dr. Rosal is a behavioral psychologist with extensive experience working with minority diabetic populations.
- The team also includes appropriately funded clinical, diabetes educator, and disparities researcher, and statistical expertise.
- Consultants experienced in Second Life application development are also secured.

Weaknesses

- None identified

3. Innovation:

Strengths

- As above.

Weaknesses

- None identified

4. Approach:

Strengths

- The RCT approach is well described and appropriate.

- The choice of a well developed, well resourced, but more traditional health education intervention of equivalent dosage and identical content is perfect for a CER project and will yield an interpretable estimation of whether Virtual reality has advantages over traditional high quality methods in this population.
- Although this proposal would only support a pilot with 50 individuals in each arm, it will cover costs of application development so that a subsequent larger intervention would have a tested intervention at the outset.

Weaknesses

- The provision of a laptop computer in the intervention group, with no equivalent for the control group could lead to greater compliance in the intervention arm. Thought should be given to whether a comparable incentive can be created – even the same laptop – for the control arm.

5. Environment:

Strengths

- The BUMS clinical environment together with its network of Boston area community clinics is well suited to providing a patient population and an appropriate intervention/analysis environment for the study.

Weaknesses

- None identified

Protections for Human Subjects:

- Acceptable: Investigators have thought through both the privacy and safety issues; seem very familiar with HIPAA requirements and experienced in recruitment and obtaining informed consent

Data and Safety Monitoring Plan (Applicable for Clinical Trials Only):

Acceptable: Although a DSMB is not mentioned, there is ample provision made for potential adverse consequences of this low risk intervention.

Inclusion of Women, Minorities and Children:

G2A - Only Women, Acceptable

M2A - Only Minority, Acceptable

C3A - No Children Included, Acceptable

- The rationale for including only African American women in this pilot is appropriated, given their high rates of physical inactivity and diabetes and their unknown but potentially high degree of appropriateness for this distance learning intervention. .

Budget and Period of Support:

Recommended budget modifications or possible overlap identified:

- Appropriate

CRITIQUE 3:

Significance: 1
Investigator(s): 1

Innovation: 1
Approach: 3
Environment: 1

Overall Impact:

Strengths

- Overall innovative project that has the potential to move the science forward.
- Shows promise to have a substantial impact on self-management behaviors for African American women, a group at especially high-risk for negative outcomes of T2DM.

Weaknesses

- Time frame may be tight and getting the project launched quickly will be imperative

1. Significance:

Strengths

- Gaps in the literature well delineated. The magnitude of the impact of diabetes as a public health problem especially the impact for women and African Americans (including double death rate in location of study for African Americans with diabetes) is well established.
- Diabetes self-management programs have been effective but have high dropout rate due to multiple family demands, accessibility, and length of program.
- Emerging studies of virtual reality have shown ability to impact behavior (including exercise) but studies are preliminary and have not addressed the spectrum of T2DM self-management behaviors.
- This study has the potential to substantially move science forward. It can make important information and development of self-management skills more accessible and potentially more effective.

Weaknesses

- None identified

2. Investigator(s):

Strengths

- Team with strong experience with this population and with low-literacy studies. Instruments validated in this population. Ability to construct this VR in SL. Together the team and the consultants have the skills and capacity to conduct the research

Weaknesses

- Large team with small % of PI in year one. This can be both a strength and weakness. The large number of team members with specific skills and tasks can be useful especially in short time line. But managing a large team with a short time line can present challenges.

3. Innovation:

Strengths

- Shifts both current research and potentially clinical practice using novel and contemporary delivery system. Translates an established self-management program aimed at African American participants to a virtual reality environment (second life -SL).

- Built on the higher than expected use of internet and technology by African American population
- Methods developed and tested in this program of research may become a model for other chronic conditions

Weaknesses

- None identified

4. Approach:

Strengths

- Pilot RCT. Limits study to urban AA women with T2DM who are physically inactive and have uncontrolled diabetes. (With women being health decision makers who can also influence others). Control group will receive the established group program (Power to Prevent) and individual counseling. The intervention group will participate in the VR program which includes the same group of individual and group activities as control group in SL environment. The program will be augmented for those who already have diabetes. The time commitment for the two programs will be approximately the same but the VR-SL group may have more, shorter sessions.
- Potential problems well thought out. For example: To minimize technical issues the intervention group will have provided equipment (with privacy mechanisms built in), access (12 months of high speed internet) , and training in accessing and using the SL platform. Training plan for SL is detailed and appropriate.
- Analysis (including dose, cost) appropriate. Instruments have been validated use low literacy populations (frequently by team). Power appropriate for aims

Weaknesses

- Analysis plan for qualitative and focus groups unclear.
- Potential weaknesses: Use of mailed letter of invitation to recruit subjects is weak and may limit ability to recruit in the timeline presented. If IRB approves telephone follow up, this mechanism should be effective. Timeline very tight. Much development activity early and intervention and data collection time might be limited. The SL intervention arm for all subjects is will be conducted in months 8-14 and eight month measures delineated for month 18.

5. Environment:

Strengths

- Large urban environment with ample population of interest. Participants will be recruited from 3 areas of the city of Boston that have the highest rates of diabetes mortality of all neighborhoods in the city. There appears to be both the personnel and environmental assets (especially in use of virtual activity) to conduct the project.

Weaknesses

- None identified

Protections for Human Subjects:

Acceptable Risks and/or Adequate Protections

- Will need to be clear what are research activities and when clinical activities (stress tests) are recommended it is under the care of their health care provider and not provided by the study.

Will need procedures for those who may become distressed during program (mental health referrals)

Data and Safety Monitoring Plan (Applicable for Clinical Trials Only):

Acceptable

No data and safety monitoring plan--no drugs and stress test under supervision of health care provider so probably do not need.

Inclusion of Women, Minorities and Children:

G2A - Only Women, Acceptable

M1A - Minority and Non-minority, Acceptable

C3A - No Children Included, Acceptable

- Focus will be on the highest risk (women and African Americans); after established in these populations study will be expanded to men, children and mixed genders)

Budget and Period of Support:

Recommended budget modifications or possible overlap identified:

- Appropriate. There is NO potential overlap with other existing grants and/or pending applications.)

THE FOLLOWING RESUME SECTIONS WERE PREPARED BY THE SCIENTIFIC REVIEW OFFICER TO SUMMARIZE THE OUTCOME OF DISCUSSSIONS OF THE REVIEW COMMITTEE ON THE FOLLOWING ISSUES:

PROTECTION OF HUMAN SUBJECTS: UNACCEPTABLE
It is not clear how patient information will be protected when using Second Life.

Data and Safety Monitoring Plan Unacceptable

There is no provision for monitoring data safety.

INCLUSION OF WOMEN PLAN (Resume): ACCEPTABLE
The study includes women only. This is acceptable.

INCLUSION OF MINORITIES PLAN (Resume): ACCEPTABLE
The degree of inclusion of minorities is appropriate.

INCLUSION OF CHILDREN PLAN (Resume): ACCEPTABLE
Children are not included. Acceptable justification for exclusion is provided.

COMMITTEE BUDGET RECOMMENDATIONS: The budget was recommended as requested.

SCIENTIFIC REVIEW OFFICER'S NOTES: An editorial board review was used to evaluate all applications submitted in response to this RFA. The roster is available at http://www.csr.nih.gov/SummaryStatementRoster/RC1_200910_ZRG1PSEJ58.pdf

Please note important information in Notice OD-09-100 (http://grants.nih.gov/grants/guide/notice-files/NOT-OD-09-100.html) about further submissions based on unsuccessful applications for American Recovery and Reinvestment Act (ARRA) Funding Opportunity Announcements.

NIH always encourages discussion with NIH Program on whether to submit an investigator-initiated application following an unsuccessful submission to an RFA. For ARRA applications this is particularly important for two reasons. First Program Officers can provide advice on if and when the application should be submitted as an R01, R21, or other type based upon the result of the review. Second the NIH Institutes and Centers included topics in ARRA announcements on a wide variety of areas that are not part of their typical scientific interests. Applications for such topics may not be suitable for investigator-initiated submissions to the NIH. Advance discussions may help avoid the submission and rejection of an inappropriate investigator-initiated application.

NOTICE: In 2008 NIH modified its policy regarding the receipt of resubmission (formerly termed amended) applications. Detailed information can be found by accessing the following URL address: http://grants.nih.gov/grants/policy/amendedapps.htm

NIH announced implementation of Modular Research Grants in the December 18, 1998 issue of the NIH Guide to Grants and Contracts. The main feature of this concept is that grant applications (R01, R03, R21, R15) will request direct costs in $25,000 modules, without budget detail for individual categories. Further information can be obtained from the Modular Grants Web site at http://grants.nih.gov/grants/funding/modular/modular.htm
