# Supplementary material for: Design and Methods for a Comparative Effectiveness Pilot Study: Virtual World vs. Face-to-Face Diabetes Self-Management
Source: JMIR Res Protoc. 2012 Dec 17;1(2):e24. doi: 10.2196/resprot.2415 (PMC3626161; doi:10.2196/resprot.2415)
Supplement: Supplementary file 3 [file resprot_v1i2e24_app3.pdf]

Response to reviewers of NIH grant application #1 RC1 LM010412-01.

A) <u>The following section A summarizes our proposed approach to address the concerns raised by the reviewers, as listed in the Resume and Summary of Discussion.</u>

A.1. Lack of Expertise in Second Life.(Summary of Discussion and reviewer 1).
<u>Response</u>: While not specifically stated in the proposal, our team in fact has extensive experience in implementation of Second Life activities, and we plan to continue to expand our portfolio of both research and applied programs in Second Life. Dr. Wiecha (study multi –PI) and the Second Life experts (Heyden and Dorland) have recently published the first careful evaluation of a medical education event in Second Life in JMIR entitled “Learning in a Virtual World: Experience With Using Second Life for Medical Education” (see: http://www.jmir.org/2010/1/e1/). The team is currently collaborating on several projects, including a WHO-funded follow-on project to augment an online professional development course on research methods, and a pilot to explore feasibility of motivational interviewing training in Second Life. We are currently developing a proposal for continuing medical education training on cancer prevention in Second Life with the California Assoc. of Family Physicians. In addition, Robin Heyden is a frequent guest speaker and conducts regular education workshops in Second Life. These workshops consist of basic building tutorials, new tools for educators, and tours of SL locations with educational relevance. Ms. Heyden has presented at a number of conferences including the Virtual Worlds Best Practices in Education conference, held in Second Life in March 2010.

A.2. Limitations in physical activity as the primary outcome (Summary of Discussion). The physical activity measure may be insufficient to detect small differences. The study seems underpowered to detect substantial improvement (in physical activity) using Second Life. Since the investigators will also be measuring hard outcomes (weight, blood pressure, glycemic indices) these should be included as primary aims. Secondary aims need to be included as primary aims. (Reviewer 1).
<u>Response</u>: We agree with the reviewers that there are limitations in our choice of physical activity as a primary outcome (Aim 2). We propose to improve our original plan by adding a 24 hour recall measure of physical activity in combination with the proposed CHAMPS questionnaire. These measures will complement each other and increase the sensitivity of our assessments. In addition, as recommended by Reviewer 1, we are now including additional outcomes as primary aims: glycemic control (HbA1c), weight, and blood pressure, While we recognize that we will not have sufficient power to detect equivalence of the study conditions on these outcomes of interest, measuring these variables will allow us to conduct exploratory analysis to estimate variance and effect size. This study will provide extremely valuable information on feasibility for a future randomized clinical trial, and the estimates of variance and effect size will be critical for the planning of sample size and power calculations of such a trial which will compare the equivalence of in-person and the Second Life interventions. Given the limited state of knowledge regarding lifestyle counseling interventions delivered within Second Life, a feasibility study is a rational first step to furthering our understanding, particularly among an underserved population proposed here. The in-person intervention condition is evidence-based and thus has shown to be effective in producing changes in the outcomes of interest, thus we expect improvements in the outcomes in this active control group. We are not proposing that the Second Life method will be superior, but rather not

inferior. As such, a future intervention will be powered on lifestyle and hard measures including glycemic control under the concept of equivalence of the effect of the in-person and the Second Life intervention conditions on the outcomes of interest. Depending on the difference in outcome that is defined to represent equivalence between 2 interventions where both are likely to produce beneficial outcomes, the sample sizes required can be very substantial in equivalence studies, thus we anticipate a much larger RCT as a follow-on study based on the results of this study, if warranted. Conversely, if we demonstrate trends indicating superiority of the SL method in this study, we would potentially power on that hypothesis in the follow-on study.

A.3. No plan for dealing with decreased adherence. Aim 2 greatly depends on Aim 1 succeeding. There is no recourse for when there is decreased adherence to the protocol. (Summary of Discussion and Reviewer 1).

Response: We appreciate the reviewers' concern regarding the importance of successfully achieving Aim 1 in order to accomplish Aim 2. Unfortunately due to space limitations we were unable to elaborate on our considerable expertise in recruiting and retaining individuals in our previous studies, including low-income and minority groups. Dr. Rosal, Ph.D., study multi-PI) has a 14-year history of research much of which has focused on health disparities, diabetes and behavior change interventions. Her two most recent studies with low-income individuals with diabetes and pre-diabetes (Latinos), achieved their recruitment goals and both had a retention rate (HbA1c at 12 months) of 93% (both studies). The screening and recruitment process as well as the retention strategies that will be implemented in the proposed study will mirror those used by Dr. Rosal in her study of diabetic Latinos.

A.4. Insufficient implementation and training plan. Implementation and training of participants is not sufficiently addressed. The digital divide is substantial in this population and might provide a substantial barrier. (Summary of Discussion & Reviewer 1).

Response: The training curriculum, outlined in the Table "Outline of Second Life Training Curriculum" in our proposal will be implemented to introduce and train subjects progressively in only those basic functions of SL needed for the study. The study staff responsible for installing the computer will begin the training process by installing the SL program and demonstrating where to locate an online training module developed by our experts. This simple online training module is self-directed. In addition, the SL experts will be available for coaching both online and via in-person home visits if necessary to bring all participants to a functional competence level in SL prior to beginning counseling activities in SL. This plan was carefully designed based on our successful experience with the project published as noted in #1 above.

We appreciate the reviewers' concerns with regard to digital divide issues, however our experience working with low income, minority inner-city populations in telemedicine projects in the past suggests that this will not be a problem in the proposed study. For example, in our pediatric asthma study which required for participants to have home computers, few subjects were excluded due to lack of home computer access.

A.5. Differential incentives for the intervention and control group (Summary of Discussion). There is a difference in incentives between intervention and control groups- the VR arm gets a MacBook at end of study. This could influence program adherence. Some comparable incentive should be considered for the control arm (Reviewer 2).

Response: We will provide the control group with a comparable computer at study start to equalize incentives between the 2 groups, since the intervention group will receive the computer at study start as well. We anticipate that most subjects will receive a computer to insure comparability of ease of internet connection and to minimize technical problems.

B) Other comments or concerns noted by the reviewers (and not listed in the Summary of Discussion) are addressed in the Section B below:

B.1. Content of Second Life is not clear; investigators need to have a clear understanding of what information will be made available and how.(Reviewer 1)
Response: The content of the curriculum, Power to Prevent, is summarized in the Table termed "Lifestyle Objectives for Real World and Virtual World Group and Individual Sessions." Page limitations precluded further description of the program. This evidence-based protocol is available at
http://www.ndep.nih.gov/publications/PublicationDetail.aspx?PubId=124
and contains discrete lessons week by week on the topics described in the above noted Table. The content of the training process for acquiring skill in using Second Life is described in the Table "Outline of Second Life Training Curriculum" and was developed directly from our experience with the project published as noted above, and also described in A.4 above.

B.2. Levels of effort proposed by investigators are unlikely to be sufficient for scope of project, raising doubts that goals will be accomplished. (Reviewer 1)
Response: We have conducted similar projects in the past and are aware that the project is ambitious. However, we will build from our experiences and experienced staff that we are able to recruit from prior studies. Our project director/coordinator will be funded at 100%, as will 2 full time recruitment staff. The coordinator will also oversee and lead the retention efforts under the close supervision and guidance of Dr. Rosal. With this infrastructure in place, we are confident that the proposed effort levels will be sufficient for the investigators.

B.3. The research assistants are going to be fully responsible for recruitment and training. They will need to be trained as well. (Reviewer 1)
Response: We have planned for the training needs of the research staff and have already begun to train the diabetes health educators in the use of Second Life, as well as the Director of Ambulatory Diabetes Services at Boston Medical Center (the supervisor of the nurse educators, and of Dr. Sternthal) who has taken a personal interest in this project.

B.4. Several web-based initiatives have been developed for diabetics. It is unclear how this one is any different.(Reviewer 1)
Response: The use of a virtual world, Second Life, is what distinguishes this intervention from prior studies. There have been no high-quality studies evaluating the impact of counseling delivered in virtual worlds on patient outcomes, and certainly no studies on particularly high-risk patients as proposed in this study. Previous educational psychology research shows that virtual worlds like Second Life offer a particular compelling sense of what has been termed "presence" for participants that promotes a deeper and more committed engagement with the activities taking place. In addition, Second Life, like other virtual worlds, were originally created as social networking environments and so are particularly conducive to promoting communication between individuals, an attribute we will feature in this program.

B.5. There is no long-term follow-up to assess sustainability, which is a major problem in this population. (Reviewer 1)
Response: This is an initial feasibility study. Should the study findings so warrant, we will build from this study to propose a larger randomized trial for a comparative effectiveness study of the in-person and Second Life interventions that will be pilot-tested in this study.

B.6. Reviewer 1 stated "It is unclear why only women are included in the study." (Reviewer 1)
Response:. Reviewer 2 listed this inclusion criteria as appropriate **.** In the proposal under "Study population" we provide the rationale for focus on AA women with diabetes: they have overall lower rates of physical activity and high rates of poorly controlled diabetes, they are the decision makers for health issues in the home thus interventions may be disseminated within their families, and research shows a strong preference among AA women for singe- sex health programs.

B.7. The analytical plan needs to be addressed for each aim. (Reviewer 1)
Response: The analytic approach for each variable will be to calculate the delta of the delta- that is, calculate for each variable the change within group from pre to post, and apply appropriate statistical testing, and then compare those changes between the 2 randomized groups. We apologize that space limitations precluded us from fully describing this analysis for each variable of interest.

B.8. There is insufficient evaluation of the Second Life implementation for this domain prior to use in the clinical trial. (Reviewer 1)
Response: We agree with the reviewer that there has been very little work in this area generally, thus the purpose of assessing the outcomes proposed in this proposal, including feasibility. We believe that this is a strength rather than a weakness of the proposed study.

B.9. There is limited information on training of patients. (Reviewer 1)
Response: Please see A.4 above.

B.10. They should not provide participants will free laptops and simply use the computers in the patient's households. (Reviewer 1)
Response: if available, we will use existing computers provided they have adequate power and speed and reliability. However, we anticipate that most subjects will receive a computer to insure comparability of ease of internet connection and to minimize technical problems. An alternative equivalent incentive will be provided in cash if subject does not require a computer to maintain equivalence of incentives across all subjects and groups.

B.11. Inadequate computers in households would present a problem in potentially expanding this project. (Reviewer 1)
Response: The digital divide will continue to narrow and the penetration of powerful personal computers and other computing devices will continue to expand. This study will help to assess the current penetration of computers into the inner-city powerful enough to run Second Life.

B.12. Second Life is proprietary and there should be more provision for security of protected health information (Reviewer 1). It is not clear how patient information will be protected when using Second Life (Reviewer 3).
Response: In terms of privacy during Second Life sessions, we will restrict access to the group sessions to only people participating in the study-- in which case, nobody else can access the area and hear or see what is happening. During the individual sessions, we can "teleport" the subject and counselor to a private platform in the virtual atmosphere above the meeting area, where nobody can see or hear the conversation, and in fact nobody else can access that area where the individual counseling is occurring because the teleportation mechanism can be inactivated by the counselor. This is akin to pulling up a ladder in a tree house. Finally, the communication between the counselor and the patient can be restricted to only those two persons, so nobody else can hear it. In addition, the consent form will list the very small risk of compromise of confidentiality during the individual sessions in Second Life, although we cannot conceive of how this might occur.

B.13. Time frame may be tight and getting the project launched quickly will be imperative. (Reviewer 3)
Response: We are confident that with the use of proven recruitment strategies and experienced staff and system infrastructure we can accomplish this project on timeline. In addition, given the new one-year timeline, we now propose delivering the 12-session intervention protocol in 5 months, rather than the 8 months originally proposed. This change will be achieved by simply providing additional group counseling sessions every week, similar to a previous pilot study of a diabetes intervention for low-income Latinos conducted by Dr. Rosal abstract available at:
http://www.ncbi.nlm.nih.gov/pubmed/15946117.
Rosal et a.. Diabetes self-management among low-income Spanish-speaking patients: a pilot study. Ann Behav Med. 2005 Jun;29(3):225-35.

B.14. Analysis plan for qualitative and focus groups is unclear. (Reviewer 3)
Response: Dr. Barnes, an expert on qualitative methods, will lead the qualitative research component of this project. Standard methods will be used. A semi-structured interview will be designed through a collaborative effort of Drs. Wiecha, Rosal and Barnes. The scope of questions will include experiences with the training and using of SL, and for both groups, experiences with the curriculum and group and individual sessions. Questions will be open-ended in nature, with pre-determined prompts to facilitate in-depth exploration of issues of interest. Participants will be recruited from both groups. They will be invited to participate in the focus groups by study staff. A total of 4 focus groups will be conducted, 2 at start to assess formative issues, and 2 at end of study – one each for face to face and SL groups. Additional focus groups may be done based on experience with the proposed 4. The groups will be led by Dr. Barnes. and an assistant. Participants will be consented prior to their participation in the groups and will receive an incentive of $ 30 for their participation. Focus groups will be audiotaped and transcribed for analysis. Analytic steps will include coding of qualitative data by two independent coders who will then collaborate in the development of a codes book. Such codes will reflect themes discussed by participants in response to the interview questions. As we proceed through our data collection and analysis, we will maintain an “audit trail,” such that someone else could, in theory, repeat each stage.

Data from the different interviews will then be excerpted by theme. The identification of themes will enable us to determine key theoretical issues, and to proceed to locate

related literature with which to analyze and compare thematic issues more deeply, i.e. theoretical frameworks. This employment of existing social science theories to analyze the data is known as theoretical triangulation.

Standard computer software will be utilized for the qualitative analyses. This software will be HyperResearch, which enables the investigators to code and retrieve, build theories, and conduct analyses of the data. It has advanced multimedia capabilities, which make it possible to work with text, graphics, audio, and video sources. It has a code-and-retrieve data analysis capacity, along with theory building features.

B.15. Use of mailed letter of invitation to recruit subjects is weak and may limit ability to recruit in the timeline presented. Timeline very tight. (Reviewer 3)
Response: This protocol has been changed to follow a direct call protocol whereby potential subjects are identified by the Boston Medical Center data warehouse analysts through automatic screening of patient data in the electronic medical record system. This process can identity potentially eligible patients in the data by gender, age, race, provider, and lab values including hemogblobin a1c values, and can exclude patients if they carry diagnoses listed in our exclusion criteria, such as cardiac conditions. Study staff will then review the electronic record for further exclusion factors, such as history of diabetic ketoacidosis, or use of steroid medication. Then the patient lists, organized by provider, will be presented to the primary care physician for approval for participation in the study. They will approve the patient for the walking activities and for use of a healthy diet. We will require HIPAA waiver, IRB approval, and permission from the healthcare providers to screen their patients to generate the provider- specific lists of potentially eligible patients. Once the patient lists are approved the subjects will be direct called by study personnel, and/or met in clinic by study personnel for recruitment activities. This method has been evaluated at BMC and published results show it to be by far the most efficient and effective method of patient recruitment in this population, compared to provider-initiated direct recruitment during clinic visits, or mailed letter recruitment. (See: Schroy et al. A Cost-effectiveness analysis of subject recruitment strategies in the HIPAA era: results from a colorectal cancer screening adherence trial. Clinical Trials 2009 Dec;6(6):597-609. Epub 2009 Nov 23).

B.16. Protection of Human Subjects. Will need to be clear what are research activities and when clinical activities (stress tests) are recommended it is under the care of their health care provider and not provided by the study.(Reviewer 3)
Response: The subjects will be informed that the study will not be paying for any treatment or screening. The primary care physician will also be informed of this at the time of referral for such testing if ordered by the PCP via direct communication with both PCP and patient by the study co-PI, Dr. Wiecha

B.17. Will need procedures for those who may become distressed during program (mental health referrals). (Reviewer 3)
Response: Because depression and emotional distress are common among patients with diabetes, all patients will be provided with mental health provider information and referral sources upon joining the study. In addition, any patient reporting undue emotional distress either during the study assessments or study intervention sessions will be encouraged to discuss his/her symptoms with the primary care provider and encouraged to request assessment and services. Protocols for the study will require staff to alert the Dr. Wiecha if any individual requests such services, and he (Dr. Wiecha) will personally make the appropriate referral through direct contact with that subject's

primary care provider.

B.18. Lack of data safety and monitoring plan (Reviewer 3).
Response: This issue was discussed with project officer, Dr. Ye who agreed that this was not necessary given the low risk characteristics of this project.
